# Oral function status of older patients seeking dental implant treatment

**DOI:** 10.1186/s40729-024-00571-w

**Published:** 2024-11-05

**Authors:** Risako Taue, Tokiko Osawa, Yoshiki Uchida, Myu Hayashi, Kentaro Kitakabu, Yuji Sato, Junichi Furuya

**Affiliations:** 1 Department of Oral Function Management, Graduate School of Dentistry, Showa University, 2-1-1 Kitasenzoku, Ota-Ku, Tokyo, 145-8515 Japan; 2 Division of Oral Function Management, Department of Oral Health Management, School of Dentistry, Showa University, 2-1-1 Kitasenzoku,Ota-ku, Tokyo, 145-8515 Japan

**Keywords:** Dental implant treatment, Older patients, Oral function, Prosthetic treatment

## Abstract

**Purpose:**

In recent years, dental implant treatment has become an option for prosthetic treatment for missing teeth and is often performed in older patients. However, the complex oral functional decline in old age presents challenges in terms of frailty prevention, making oral function management after prosthetic treatment crucial. Nonetheless, the actual status of oral function in older patients seeking dental implant treatment remains unclear. In this study, we aimed to assess the oral function status of older patients seeking dental implant treatment.

**Methods:**

Among patients receiving prosthetic treatment for missing teeth, 227 older patients (111 in the pre-dental implant group and 116 in the pre-bridge/denture group) who underwent a thorough examination of their oral function were included in this study. Age, sex, comorbidities, smoking status, number of functional teeth, and occlusal support status were obtained from the medical records. Statistical analyses were performed using the t-test, chi-square test, and logistic regression (p < 0.05).

**Results:**

Compared with the pre-bridge/denture group, the pre-dental implant group had significantly better oral hygiene, occlusal force, tongue-lip motor function, tongue pressure, masticatory function, and swallowing function and a significantly lower prevalence of oral hypofunction. Older age and decreased occlusal support were associated with the diagnosis of oral hypofunction, even after adjusting for confounding factors including prosthetic treatment.

**Conclusions:**

Although older patients seeking dental implant treatment have a higher oral function than those seeking general prosthetic treatment, older age and a lower number of occlusal supports suggest that appropriate oral function management is needed.

## Background

The global population is aging at an accelerating pace [1]. In Japan, the percentage of the total population aged 65 and over (the aging rate) was 23.0% in 2010 and 28.9% in 2021, and it is expected to reach 38% by 2065, indicating the rapid aging of the population [2, 3]. In 2021, the proportion of older people aged 75 years or older accounted for more than 1/8 of the total population [3]. Currently, the rate of elderly people requiring support or care in the later stages of life is 38.1%, as reported by physicians, and they are expected to extend their healthy life expectancy and be independent both physically and mentally. However, there is a large discrepancy between average life expectancy and healthy life expectancy, it is therefore important to prevent frailty for reducing this gap [4]. Frailty is a state of increased physical and mental fragility, and the increase in the number of frail older individuals is directly related to the increase in the number of older individuals requiring nursing care [5]. Progressive frailty is often related to dietary issues, which are caused by a complex decline in oral functions (oral frailty) [6]. Indeed, complex oral function decline is associated with future deterioration in physical and mental functions [7], and integrated oral function management is important for preventing frailty [8]. To manage the complex decline of oral function in old age, the Japanese Society of Geriatric Dentistry proposed the term “oral hypofunction” in 2016, which was incorporated into public insurance in 2018 [9]. Oral hypofunction is a composite evaluation of oral functions, consisting of seven items [9]. Poor oral function is associated with sarcopenia [10], malnutrition [11], mild dementia [12], frailty [8, 13, 14], and health-related quality of life [15]. Moreover, oral function declines in complex ways in older individuals [8, 16] suggesting the importance of appropriate oral function management for oral hypofunction in old age.

Previous studies reported an increase in dental implant treatment in older individuals as an option for prosthetic defects [17, 18]. Thus, it is expected that there will be increasing opportunities to provide dental implant treatment to older patients with oral hypofunction. Therefore, managing post-treatment oral function in older patients seeking dental implants would be crucial to preventing frailty and extending healthy life expectancy. However, the actual state of oral hypofunction in patients seeking dental implant treatment remains unclear. Therefore, we hypothesized that older patients seeking dental implant treatment would need oral function management even though they have better oral function than older patients seeking bridge or denture treatment. This study aimed to compare the comprehensive oral function of older outpatients who requested prosthetic treatment with either dental implants or bridges/dentures, to clarify the actual condition of oral function in older patients who received dental implant treatment.

## Methods

### Study participants

**Fig. 1:**
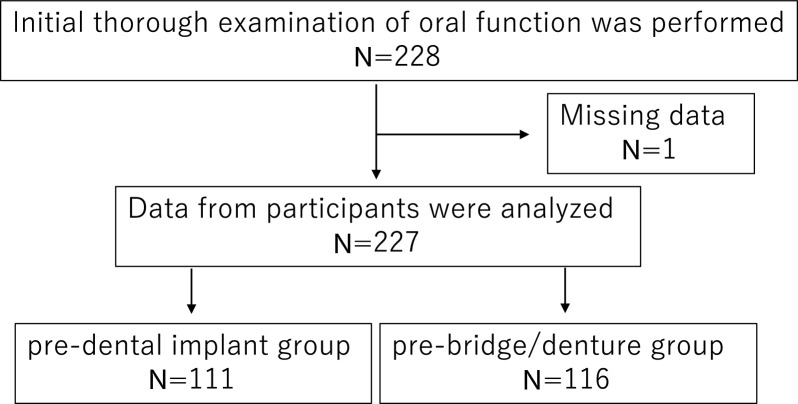
Participant selection flowchart

This study included 228 patients, aged ≥ 65 years, who visited the Department of Oral Function Management, Showa University Dental Hospital, between June 2019 and December 2023 for an initial thorough oral function examination. The exclusion criteria were incomplete data and lack of consent for examination. Finally, 227 patients participated, of whom 111 and 116 were classified into the pre-dental implant group (those desiring dental implant treatment) and pre-bridge/denture group (those desiring prosthetic treatment other than dental implants) (Fig. 1). Consent was obtained from the study participants using the opt-out method. After a detailed explanation of the treatment options by the dentist in charge, the patient consulted with the dentist and selected his or her own treatment plan. This study was approved by the Ethical Review (DH2018-032).

### Study methodology

Data on age, sex, comorbidities, smoking status, number of functional teeth, occlusal support status, oral hypofunction [encompassing oral hygiene, oral dryness, occlusal force, tongue-lip motor function (oral diadochokinesis), tongue pressure, masticatory function, and swallowing function], and the applicability of the diagnosis of oral hypofunction were collected from the patients’ medical records. Comorbidities were assessed using the Charlson Comorbidity Index (CCI) [19]. The presence or absence of psychiatric disorders other than hypertension and dementia, which are not included in the CCI but are common among the older and likely affect oral function (e.g., smoking), was also investigated. The number of remaining natural teeth, excluding those with mobility degree III and caries degree IV, and the number of functional teeth, which included remaining natural teeth plus prosthetic teeth (including pontics of bridges, dentures, and dental implants), were recorded. The occlusal support status, including the final dental implant prosthesis and pontics of already placed bridges, was evaluated using the Eichner classification. This classification, defined as a modified version of the Eichner classification, was applied to assess the occlusal support status.

Oral hypofunction was evaluated and diagnosed based on established guidelines [9]. Oral function was assessed and diagnosed by several well-trained and qualified dentists. All evaluators received 2 h of lecture and 2 h of hands-on training according to the manual to ensure consistency and accuracy in their evaluations. Oral hygiene was visually assessed to determine the degree of tongue coating using the Tongue Coating Index. The tongue surface was divided into nine sections, and the degree of tongue coating was evaluated at three levels (0, 1, 2) for each area. The sum of the scores for all areas ≥ 50% indicated poor oral hygiene. Oral dryness was assessed as the degree of mucosal wetness at the center of the dorsum of the tongue, approximately 10 mm from the apex of the tongue. An oral cavity moisture tester (Mucus; LIFE Corp., Saitama, Japan) was used for the measurements, and a special sensor cover was applied to the sensor to ensure uniform pressure contact. Measurements were performed thrice, and the median value was used for evaluation: a median value < 27.0 indicated oral dryness. Occlusal forces were measured using a pressure-sensitive sheet Dental Prescale II; GC (Dental Prescale II; GC Corp., Tokyo, Japan) and an analyzer (Bite Force Analyzer; GC Corp., Tokyo, Japan) for the entire dentition during clenching for 3 s in the occlusal–occipital fit position. A bite force < 500 N indicated reduced occlusal force. Tongue-lip motor functions were examined by asking patients to repeatedly pronounce /pa/, /ta/, and /ka/, and the number of repetitions per second was measured using an automatic measuring device (Kenko-kun Handy; Takei Kikai Kogyo Corp., Niigata, Japan). Patients were classified as having decreased tongue-lip motor functions if either repetition was < 6 per second. Tongue pressure was measured using a tongue pressure measuring device (JMS Corp., Hiroshima, Japan). Dentures were worn during the measurement. A maximum tongue pressure < 30 kPa indicates decreased tongue pressure. Masticatory function was assessed by measuring glucose concentration after chewing a gummy jelly. After 2 g of gummy jelly (Glucolam; GC Corp., Tokyo, Japan) was chewed for 20 s, 10 mL of water was added, and the gummy and water were discharged through a filter mesh. A glucose concentration < 100 mg/dL indicates decreased masticatory function. Swallowing function was assessed using a swallowing screening questionnaire (The 10-item Eating Assessment Tool). A total score ≥ 3 points indicates a deterioration in swallowing function. Oral hypofunction was diagnosed based on factors such as poor oral hygiene, oral dryness, reduced occlusal force, decreased tongue-lip motor function, reduced tongue pressure, decreased masticatory function, or deterioration in swallowing function, with a diagnosis made if three or more of these seven factors were present.

### Statistical analyses

The t-test was used to compare the means of each of the seven continuous variables that were normalized using the Shapiro–Wilk test: age, CCI score, number of functional teeth, and means of each of the seven items in the detailed examination of oral function between the pre-implant and pre-bridge/denture treatment groups. Categorical variables, sex, occlusal support status, hypertension, mental disease, and smoking status were compared between the pre-implant and pre-bridge/denture groups using the chi-square test. Logistic regression analysis was conducted to clarify which factors were related to the presence or absence of a diagnosis of poor oral function and the presence or absence of each of the seven items of the detailed oral function test as objective variables. All statistical analyses were performed using IBM SPSS Statistics version 25.0 (IBM, Armonk, NY, USA), with a significance level of 5%.

## Results

### Patient characteristics

Table 1 summarizes the patient characteristics. Significant differences in age, CCI score, number of functional teeth, sex, occlusal support status, and hypertension were observed between the pre-dental implant and pre-bridge/denture groups.

**Table 1:** Comparison of demographic and clinical characteristics between pre-dental implant and pre-bridge/denture groups

Characteristic	Pre-dental implant group (N = 111)	Pre-bridge/denture group (N = 116)	P-value
Age (years)	73 (69–77) 73.1 (5.4)	80 (75–84) 79.8 (6.7)	< 0.001*
CCI	0 (0–0) 0.3 (0.7)	0 (0–1) 0.8 (1.1)	0.013*
Functional teeth	25 (23–26) 24.4 (2.6)	28 (25–28) 26.1 (3.8)	0.003*
Sex			0.041**
Male	36 (32.4%)	53 (45.7%)	
Female	75 (67.6%)	63 (54.3%)	
Occlusal support status			< 0.001**
A group	33 (29.7%)	9 (7.8%)	
B group	75 (67.6%)	63 (54.3%)	
C group	3 (2.7%)	44 (37.9%)	
Hypertension	42 (37.8%)	64 (55.2%)	0.009**
Mental disease	0 (0.0%)	2 (1.7%)	0.165
Smoking	0 (0.0%)	3 (2.6%)	0.088

Data presented as median (Q1–Q3), mean (SD), or N (%)

*CCI* Charlson’s Comorbidity Index, *SD* standard deviation

Significance levels: p < 0.05 *(t-test); p < 0.05** (Chi-squared test)

The average patient age was 73.1 years in the pre-dental implant group and 79.8 years in the pre-bridge/denture group, with patients in the pre-dental implant group being significantly younger and more likely to be female. The patients in the pre-dental implant group had lower total CCI scores and lower incidence of hypertension than those in the pre-bridge/denture group. The occlusal support status was higher in the pre-dental implant group in the order of modified Eichner classification groups B, A, and C, whereas it was higher in the pre-bridge/denture group in the order of groups B, C, and A. The total CCI score was lower in the pre-dental implant group than in the pre-bridge/denture group. The number of functional teeth was higher in the pre-bridge/denture group than in the pre-dental implant group.

Table 2 shows the mean values of the seven detailed oral function tests for each group and the percentage of patients diagnosed with oral cavity hypofunction.

**Table 2:** Comparison of oral function tests between pre-dental implant and pre-bridge/denture groups

Oral function	Pre-dental implant group	Pre-bridge/denture group	P-value
Poor oral hygiene (mean SD)	4.8 (± 3.2)	6.0 (± 4.4)	0.016*
Oral dryness (mean SD)	28.9 (± 3.0)	28.9 (± 4.3)	0.947
Reduced occlusal force (mean SD)	630.4 (± 329.4)	405.7 (± 311.7)	< 0.001*
Decreased tongue-lip motor function /pa/ (mean SD)	6.3 (± 0.7)	5.7 (± 1.0)	< 0.001*
Decreased tongue-lip motor function /ta/ (mean SD)	6.4 (± 0.8)	5.7 (± 0.9)	< 0.001*
Decreased tongue-lip motor function /ka/ (mean SD)	5.9 (± 0.7)	5.2 (± 1.0)	< 0.001*
Decreased tongue pressure (mean SD)	30.5 (± 7.2)	27.5 (± 7.8)	0.003*
Decreased masticatory function (mean SD)	165.8 (± 58.8)	116.7 (± 57.6)	< 0.001*
Deterioration of swallowing function (mean SD)	1.0 (± 2.6)	2.4 (± 5.60)	0.019*
Oral hypofunction	41 (36.9%)	83 (71.6%)	< 0.001**

*SD* standard deviation

Significance levels: p < 0.05 *(t-test); p < 0.05** (Chi-squared test)

In the pre-dental implant group, the mean values of decreased tongue-motor functions for /ka/ were lower than the reference values. In contrast, the mean values of occlusal force, tongue-lip motor functions for /pa/, /ta/, and /ka/, and tongue pressure were lower than the reference values in the pre-bridge/denture group. Compared to the pre-bridge/denture group, the pre-dental implant group had significantly higher scores for the diagnosis of poor oral hygiene, reduced occlusal force, decreased tongue-lip motor function /pa/, /ta/, and /ka/, decreased tongue pressure, decreased masticatory function, deterioration of swallowing function, and oral hypofunction.

### Diagnosis of oral hypofunction

Table 3 presents the diagnoses of oral cavity hypofunction. Logistic regression analysis was performed using the presence or absence of oral cavity hypofunction as an objective variable.

**Table 3:** Logistic regression analysis for predictors of oral hypofunction

Explanatory variables	Crude odds ratio		Adjusted odds ratio	
	95% CI	P-value	95% CI	P-value
Prosthetic details	4.294 [2.458–7.502]	< 0.001*	1.893 [0.867–3.902]	0.112
Age	1.124 [1.076–1.174]	< 0.001*	1.090 [1.036–1.146]	0.001*
Sex (0 = male, 1 = female)	1.047 [0.631–1.788]	0.866	1.354 [0.722–2.540]	0.345
Functional teeth	0.981 [0.910–1.057]	0.613	0.967 [0.877–1.066]	0.499
Occlusal support status	3.446 [2.092–5.678]	< 0.001*	2.415 [1.361–4.285]	0.003*
CCI	1.258 [0.913–1.732]	0.161	1.133 [0.788–1.630]	0.500
Hypertension (1 = positive)	1.929 [1.133–3.284]	0.016*	1.535 [0.832–2.829]	0.170
Mental disease (1 = positive)	0.829 [0.051–13.423]	0.829	0.755 [0.040–14.88]	0.866
Smoking (1 = positive)	0.411 [0.037–4.593]	0.470	0.205 [0.014–2.977]	0.246

*CCI* Charlson’s Comorbidity Index, *CI* confidence interval

Significance levels: p < 0.05* (logistic regression analysis). The objective variable is diagnosis of oral hypofunction (normal oral function = 0, oral hypofunction = 1). Among the explanatory variables, age, functional teeth, occlusal support status, and CCI are continuous variables. Prosthetic details (pre-dental implant group = 0, bridge and denture pre-treatment group = 1). Occlusal support status (modified Eichner’s classification: A group = 1, B group = 2, C group = 3)

The explanatory variables included prosthetic defects (pre-dental implant group, pre-bridge/denture group), age, sex, number of functional teeth, occlusal support status, CCI score, hypertension, mental illness, and smoking status. The results showed that older age and fewer occlusal supports were significantly associated with the diagnosis of oral hypofunction, even after adjusting for confounding factors.

### Each test item in the detailed examination of oral function

Table 4 presents the objective and explanatory variables that were significantly associated with the results of the logistic regression analysis, with the presence or absence of decline in each test item included as the objective variable. The explanatory variables included prosthetic content, age, sex, number of functional teeth, occlusal support status, CCI score, hypertension, psychiatric disorders, and smoking status. Oral hygiene in the pre-dental implant group was less likely to have poor oral hygiene compared to those in the pre-bridge/denture group. No items showed a significant association with oral dryness. Decreased occlusal force was less commonly observed in the pre-dental implant group than in the pre-bridge/denture group. However, regardless of the type of prosthetic treatment, a reduction in the number of occlusal supports was associated with a higher likelihood of experiencing decreased occlusal force. Older age and male sex were more likely to be associated with decreased tongue and lip motor functions for /pa/, /ta/, and /ka/. Additionally, a reduction in the number of occlusal supports was associated with a higher likelihood of decreased tongue and lip motor functions for /ta/ and /ka/. Regardless of the type of prosthetic treatment, older age was associated with a higher likelihood of lower tongue pressure. The patients in the pre-dental implant group were less likely to experience a decline in masticatory ability compared to those in the pre-bridge/denture group. In addition, regardless of the type of prosthetic treatment, a reduction in the number of occlusal supports was associated with a higher likelihood of experiencing a decline in masticatory ability. No items showed a significant association with decreased swallowing function.

**Table 4:** Logistic regression analysis for specific oral functions

Object variable	Significant explanatory variables	Crude odds ratio		Adjusted odds ratio	
		95% CI	P-value	95% CI	P-value
Poor oral hygiene	Prosthetic details	3.368 [1.732–6.552]	< 0.001*	4.200 [1.814–9.726]	0.001*
Reduced occlusal force	Prosthetic details	3.151 [1.824–5.444]	< 0.001*	2.277 [1.094–4.738]	0.028*
	Occlusal support status	3.249 [1.985–5.319]	< 0.001*	2.467 [1.429–4.258]	0.001*
Decreased tongue-lip motor function /pa/	Age	1.131 [1.082–1.181]	< 0.001*	1.109 [1.055–1.167]	< 0.001*
	Sex	0.432 [0.250–0.748]	0.003*	0.429 [0.231–0.796]	0.007*
Decreased tongue-lip motor function /ta/	Age	1.152 [1.100–1.207]	< 0.001*	1.133 [1.075–1.194]	< 0.001*
	Sex	0.432 [0.250–0.748]	0.003*	0.400 [0.210–0.763]	0.005*
	Occlusal support status	2.442 [1.534–3.887]	< 0.001*	1.796 [1.019–3.165]	0.043*
Decreased tongue-lip motor function /ka/	Age	1.137 [1.086–1.191]	< 0.001*	1.117 [1.059–1.178]	< 0.001*
	Sex	0.426 [0.241–0.754]	0.003*	0.419 [0.218–0.804]	0.009*
	Occlusal support status	2.924 [1.802–4.745]	< 0.001*	2.412 [1.336–4.354]	0.003*
Decreased tongue pressure	Age	1.091 [1.048–1.136]	< 0.001*	1.083 [1.035–1.133]	0.001*
Decreased masticatory function	Prosthetic details	4.056 [2.097–7.848]	< 0.001*	4.024 [1.677–9.658]	0.002*
	Occlusal support status	3.262 [1.906–5.582]	< 0.001*	2.426 [1.314–4.517]	0.005*

Significance levels: p < 0.05* (logistic regression analysis). The objective variable is diagnosis of oral function (normal oral function = 0, oral hypofunction = 1). Explanatory variables included prosthetic details, age, sex, functional teeth, occlusal support status, CCI, hypertension, mental disease, and smoking. Among the explanatory variables, age, functional teeth, occlusal support status, and CCI are continuous variables. Prosthetic details (pre-dental implant group = 0, bridge and denture pre-treatment group = 1). Occlusal support status (modified Eichner’s classification: A group = 1, B group = 2, C group = 3)

*CCI* Charlson’s Comorbidity Index, *CI* confidence interval

## Discussion

This study analyzed the differences in oral function and overall health status between the pre-dental implant and pre-bridge/denture groups in older people. Compared with the pre-bridge/denture group, the pre-dental implant group showed significantly better oral hygiene, occlusal force, tongue and lip motor function, tongue pressure, masticatory function, and swallowing function. Additionally, the prevalence of oral function decline was significantly lower in the pre-dental implant group than in the pre-bridge/denture group. However, despite adjusting for confounding factors, older age and a decrease in occlusal support were still associated with oral functional decline. The number of older people seeking implant treatment is increasing [18]. It is expected that the number of healthy older people will continue to increase as healthy life expectancy increases [3], and thus the number of older people seeking implant treatment will also continue to increase. Older patients who seek implant treatment generally have better overall oral function than those who seek conventional prosthetic treatment, but if they are older or have reduced occlusal support, it is important to combine implant treatment with appropriate oral function management based on the leaflet issued by the Japanese Society of Gerodontics, “Oral Function For those who have been diagnosed with poor oral function”. In older patients, age-appropriate targets should be set, and if a decline in occlusal strength is observed, dental treatment, encouragement of a more satisfying diet, and instruction in masticatory muscle training should be provided.

The mean values of the seven detailed oral function tests for each group, along with the percentage of patients diagnosed with oral hypofunction, suggest that patients seeking dental implant treatment are less likely to experience a decline in complex oral functions. Additionally, as decreased tongue and lip motor functions for /ka/ are more prone to decline than for /pa/ and /ta/ [20, 21], even in the pre-dental implant group, the average value for tongue and lip motor functions for /ka/ was lower than the reference value. Age, Eichner classification, and neurodegenerative diseases are relevant for diagnosing oral function decline [22]. Therefore, these confounding factors were adjusted for in the analysis. Regardless of whether the patients opted for dental implant or bridge or denture treatment, older age and a decrease in occlusal support were significantly associated with oral functional decline. This suggests that, particularly for patients with these characteristics, it is essential to provide appropriate oral function management, even for those undergoing dental implant treatment. Age, Eichner classification, and neurodegenerative diseases are associated with the diagnosis of oral function decline [22]. Our study results support these findings. Older individuals are more likely to experience a decline in oral function due to aging and disease [8, 16], with a particular tendency toward decreased tongue pressure, masticatory ability, and swallowing function as age progresses. Specifically, a decrease in occlusal support is significantly associated with a decline in masticatory ability. To maintain masticatory function in older individuals, it is important to prevent the collapse of occlusal support in the posterior teeth [23]. In our study, the patients in the pre-dental implant group exhibited higher oral literacy compared to the patients in the pre-bridge/denture group. Implant treatment was desired by patients with higher incomes [24], who had regular dental visits [25], suggesting that they were more oral-literate. Oral hygiene management significantly affects the success rate and long-term prognosis of dental implant treatment. The success rate of the treatment in older people also requires an understanding of psychological aspects [26]. Therefore, a high level of oral literacy and the dentist’s understanding of the patient’s psychological aspects are important for patients seeking this type of treatment. The lack of a significant association with oral dryness is consistent with previous reports indicating that salivary gland function is well-maintained in healthy older individuals [27]. This outcome is likely influenced by the fact that our study patients were relatively healthy, older individuals who were able to attend outpatient clinics. The results regarding occlusal force and masticatory function are attributable to the fact that the pre-bridge/denture group, which included many patients with dentures, often has insufficient occlusal support. Regarding the decline in tongue and lip motor functions, the results showed that oral perioral muscles deteriorate with age and that men are more likely to experience this decline than women. These findings support previous study results [28]. Additionally, a reduction in the number of occlusal supports is associated with the motor function of the anterior (/ta/) and posterior (/ka/) parts of the tongue.

Because this study focused on the number of occlusal supports in the posterior teeth, a reduction in the frequency of chewing in the posterior region may lead to disuse-related deterioration of tongue function. Lower tongue pressure was more likely to occur with increasing age, which is consistent with the results of previous studies [16]. A decrease in tongue pressure increases the risk of swallowing disorders. Therefore, monitoring tongue function over time is crucial in older individuals [29]. The lack of significant associations with decreased swallowing function is suggested to be due to the lower prevalence of swallowing function decline compared to the other six items [21] and the fact that the study patients, who were able to attend outpatient clinics, had a lower prevalence of swallowing function decline.

The limitations of this study include a small sample size and the single-center design, which may have introduced bias into the data. In the present study, we do not know the extent to which values were consistent among examiners. However, we believe that the assessment of oral function is a quantitative method of examination based on objective criteria and, moreover, it was performed by multiple trained and qualified dentists to minimize variability. The present study was a cross-sectional study before prosthetic treatment, but we are currently conducting a prospective study after prosthetic treatment. In the future, we would like to clarify how implant treatment changes oral function in elderly patients.

## Conclusions

Older patients seeking dental implant treatment generally maintain better overall oral function than those seeking conventional prosthetic treatments. However, providing appropriate oral function management alongside dental implant treatment is considered essential if they have lost occlusal support.

## Data Availability

The data that support the findings of this study are available from the corresponding author, upon reasonable request.

## Abbreviations

CCI: Charlson’s Comorbidity Index
CI: Confidence interval
SD: Standard deviation

## References

[1] United Nations. World population prospects 2022: summary of results. https://www.un.org/development/desa/pd/sites/www.un.org.development.desa.pd/files/wpp2022_summary_of_results.pdf. Accessed 15 July 2024.

[2] Ministry of Health, Labour and Welfare. Overview of the system and the basic statistics. https://www.mhlw.go.jp/english/wp/wp-hw14/dl/01e.pdf. Accessed 7 Oct 2024.

[3] Cabinet Office Japan. Annual report on the ageing society [summary] FY 2022. https://www8.cao.go.jp/kourei/english/annualreport/2022/pdf/2022.pdf. Accessed 7 Oct 2024.

[4] Hosokawa R, Ojima T, Myojin T, Kondo K, Kondo N. Geriatric symptoms associated with healthy life expectancy in older people in Japan. Environ Health Prev Med. 2023;28:44. 37423739 10.1265/ehpm.22-00300 PMC10331002

[5] Fukutomi E, Okumiya K, Wada T, et al. Relationships between each category of 25-item frailty risk assessment (Kihon checklist) and newly certified older adults under long-term care insurance: a 24-month follow-up study in a rural community in Japan. Geriatr Gerontol Int. 2015;15:864–71. 25316532 10.1111/ggi.12360

[6] Motokawa K, Mikami Y, Shirobe M, et al. Relationship between chewing ability and nutritional status in Japanese older adults: a cross-sectional study. Int J Environ Res Public Health. 2021;18:1216. 33572969 10.3390/ijerph18031216 PMC7908427

[7] Tanaka T, Takahashi K, Hirano H, et al. Oral frailty as a risk factor for physical frailty and mortality in community-dwelling elderly. J Gerontol A Biol Sci Med Sci. 2018;73:1661–7. 29161342 10.1093/gerona/glx225

[8] Watanabe Y, Hirano H, Arai H, et al. Relationship between frailty and oral function in community-dwelling elderly adults. J Am Geriatr Soc. 2017;65:66–76. 27655106 10.1111/jgs.14355

[9] Minakuchi S, Tsuga K, Ikebe K, et al. Oral hypofunction in the older population: position paper of the Japanese society of gerodontology in 2016. Gerodontology. 2018;35:317–24. 29882364 10.1111/ger.12347

[10] Kugimiya Y, Iwasaki M, Ohara Y, et al. Relationship between oral hypofunction and sarcopenia in community-dwelling older adults: the Otassha study. Int J Environ Res Public Health. 2021;18:6666. 34205795 10.3390/ijerph18126666 PMC8296410

[11] Iwasaki M, Motokawa K, Watanabe Y, et al. Oral hypofunction and malnutrition among community-dwelling older adults: evidence from the Otassha study. Gerodontology. 2022;39:17–25. 34212426 10.1111/ger.12580

[12] Nakamura M, Hamada T, Tanaka A, et al. Association of oral hypofunction with frailty, sarcopenia, and mild cognitive impairment: a cross-sectional study of community-dwelling Japanese older adults. J Clin Med. 2021;10:1626. 33921265 10.3390/jcm10081626 PMC8068799

[13] Shimazaki Y, Nonoyama T, Tsushita K, Arai H, Matsushita K, Uchibori N. Oral hypofunction and its association with frailty in community-dwelling older people. Geriatr Gerontol Int. 2020;20:917–26. 32830417 10.1111/ggi.14015

[14] Watanabe Y, Okada K, Kondo M, Matsushita T, Nakazawa S, Yamazaki Y. Oral health for achieving longevity. Geriatr Gerontol Int. 2020;20:526–38. 32307825 10.1111/ggi.13921

[15] Cho MJ, Kim EK. Subjective chewing ability and health-related quality of life among the elderly. Gerodontology. 2019;36:99–106. 30565311 10.1111/ger.12385

[16] Kugimiya Y, Watanabe Y, Ueda T, et al. Rate of oral frailty and oral hypofunction in rural community-dwelling older Japanese individuals. Gerodontology. 2020;37:342–52. 32141117 10.1111/ger.12468

[17] Schimmel M, Müller F, Suter V, Buser D. Dental implants for elderly patients. Periodontol. 2000;2017(73):228–40. 10.1111/prd.12166 28000268

[18] Sato Y, Kitagawa N, Isobe A. Dental implant treatment in ultra-aged society. Jpn Dent Sci Rev. 2018;54:45–51. 29755614 10.1016/j.jdsr.2017.12.002 PMC5944061

[19] Charlson ME, Pompei P, Ales KL, MacKenzie CR. A new method of classifying prognostic comorbidity in longitudinal studies: development and validation. J Chronic Dis. 1987;40:373–83. 3558716 10.1016/0021-9681(87)90171-8

[20] Hatanaka Y, Furuya J, Sato Y, et al. Impact of oral health guidance on the tongue-lip motor function of outpatients at a dental hospital. Gerodontology. 2022;39:83–9. 34689371 10.1111/ger.12599 PMC9298372

[21] Hatanaka Y, Furuya J, Sato Y, et al. Associations between oral hypofunction tests, age, and sex. Int J Environ Res Public Health. 2021;18:10256. 34639564 10.3390/ijerph181910256 PMC8508206

[22] Onuki W, Magara J, Tsujimura T, et al. Survey of oral hypofunction in older outpatients at a dental hospital. J Oral Rehabil. 2021;48:1173–82. 34346106 10.1111/joor.13237

[23] Higashi K, Hatta K, Mameno T, et al. The relationship between changes in occlusal support and masticatory performance using 6-year longitudinal data from the SONIC study. J Dent. 2023;139: 104763. 37879558 10.1016/j.jdent.2023.104763

[24] Abbas H, Aida J, Saito M, et al. Income or education, which has a stronger association with dental implant use in elderly people in Japan? Int Dent J. 2019;69:454–62. 31250446 10.1111/idj.12491 PMC9378978

[25] Tu RY, Liang P, Tan AJ, et al. Factors associated with regular dental attendance by aged adults: a systematic review. Gerodontology. 2023;40:277–87. 36271656 10.1111/ger.12661

[26] Seki K, Ikeda T, Urata K, Shiratsuchi H, Kamimoto A, Hagiwara Y. Correlations between implant success rate and personality types in the older people: a preliminary case control study. J Dent Sci. 2022;17:1266–73. 35784148 10.1016/j.jds.2021.11.014 PMC9236896

[27] Astor FC, Hanft KL, Ciocon JO. Xerostomia: a prevalent condition in the elderly. Ear Nose Throat J. 1999;78:476–9. 10429321

[28] Min SY, Pang NS, Kim YR, Jeong SA, Jung BY. Factors associated with age-related changes in oral diadochokinesis and masticatory function in healthy old adults. BMC Oral Health. 2024;24:462. 38627762 10.1186/s12903-024-04214-9 PMC11020950

[29] Sakamoto Y, Oyama G, Umeda M, et al. Effect of decreased tongue pressure on dysphagia and survival rate in elderly people requiring long-term care. J Dent Sci. 2022;17:856–66. 35756815 10.1016/j.jds.2021.09.031 PMC9201529

